# Real-world testing of an artificial intelligence algorithm for the analysis of chest X-rays in primary care settings

**DOI:** 10.1038/s41598-024-55792-1

**Published:** 2024-03-03

**Authors:** Queralt Miró Catalina, Josep Vidal-Alaball, Aïna Fuster-Casanovas, Anna Escalé-Besa, Anna Ruiz Comellas, Jordi Solé-Casals

**Affiliations:** 1 Unitat de Suport a la Recerca de la Catalunya Central, Fundació Institut Universitari per a la Recerca a l’Atenció Primària de Salut Jordi Gol i Gurina, Sant Fruitós de Bages, Spain; 2 Health Promotion in Rural Areas Research Group, Gerència d’Atenció Primària i a la Comunitat de la Catalunya Central, Institut Català de la Salut, Carrer Pica d’Estats, 13-15, 08272 Sant Fruitós de Bages, Barcelona Spain; 3 Faculty of Science Technology and Engineering, University of Vic-Central University of Catalonia, Vic, Spain; 4 Faculty of Medicine, University of Vic-Central University of Catalonia, Vic, Spain; 5 Data and Signal Processing Group, Faculty of Science, Technology and Engineering, University of Vic-Central University of Catalonia, Vic, Spain; 6 Department of Psychiatry, University of Cambridge, Cambridge, UK

**Keywords:** Health care, Medical research

## Abstract

Interpreting chest X-rays is a complex task, and artificial intelligence algorithms for this purpose are currently being developed. It is important to perform external validations of these algorithms in order to implement them. This study therefore aims to externally validate an AI algorithm’s diagnoses in real clinical practice, comparing them to a radiologist’s diagnoses. The aim is also to identify diagnoses the algorithm may not have been trained for. A prospective observational study for the external validation of the AI algorithm in a region of Catalonia, comparing the AI algorithm’s diagnosis with that of the reference radiologist, considered the gold standard. The external validation was performed with a sample of 278 images and reports, 51.8% of which showed no radiological abnormalities according to the radiologist's report. Analysing the validity of the AI algorithm, the average accuracy was 0.95 (95% CI 0.92; 0.98), the sensitivity was 0.48 (95% CI 0.30; 0.66) and the specificity was 0.98 (95% CI 0.97; 0.99). The conditions where the algorithm was most sensitive were external, upper abdominal and cardiac and/or valvular implants. On the other hand, the conditions where the algorithm was less sensitive were in the mediastinum, vessels and bone. The algorithm has been validated in the primary care setting and has proven to be useful when identifying images with or without conditions. However, in order to be a valuable tool to help and support experts, it requires additional real-world training to enhance its diagnostic capabilities for some of the conditions analysed. Our study emphasizes the need for continuous improvement to ensure the algorithm’s effectiveness in primary care.

## Introduction

Radiology is the speciality of medicine that uses different imaging techniques to detect and treat diseases. One of the most widely used imaging techniques in this field is radiography (X-ray), used to generate images of the inside of the body, which has the advantage of being quick to perform and requiring low levels of radiation^1,2^. Within the scope of X-rays, one of the most common tests is the chest X-ray^3–6^, used to detect pulmonary and cardiovascular diseases, among others.

Despite the importance of this speciality and the volume of radiological tests that exist, the lack of radiologists able to interpret them^7–11^, as well as the increase in errors in interpretation as a consequence of a heavy workload is becoming increasingly evident^12–15^. Furthermore, radiological diagnosis is a component of clinical competencies in various specialities, including family and community medicine. It is a common practice for practitioners in this field to interpret chest X-rays, despite their lower degree of expertise^16^. This fact highlights the need and, therefore, the opportunity to introduce tools such as artificial intelligence (AI) into this field to support radiologists and other healthcare practitioners who need to interpret an X-ray.

Technological support tools, such as computer-aided diagnostics (CAD), have long been used in this area. However, the advent of deep learning and machine learning models in recent years has offered the potential to develop new support tools aiming to overcome the primary limitations of CAD and enhance accuracy^17,18^. Deep learning models, compared to CAD, are built to train and work with large databases, to have constant improvement over time by learning from errors, and to have the ability to detect more than one condition at a time, all of which makes them much more powerful than CAD.

In this context, AI algorithms, including deep and machine learning models can assist in diagnosis, potentially enhancing diagnostic accuracy. However, while AI will not replace professionals, it is essential to acknowledge that the implementation of AI in routine clinical practice must be both safe and effective^20,21^. One of the current concerns lies in the fact that many of the studies on applications of new AI models only present in silico validation, a phenomenon called “digital exceptionalism”, without performing external validation in the actual implementation environment. External validation is important, as it allows estimating the accuracy of the model in a population different from the training population, selected in real clinical practice, thus allowing the subsequent generalisation of the results^22–26^.

A recent study conducting external validation of an AI algorithm designed to classify chest X-rays as normal or abnormal using a cohort of real images from two primary care centres, indicated that additional training with data from these environments was required to enhance the algorithm’s performance, particularly in clinical setting different from its initial training environment^27^.

In this context, external validation, crucial for ensuring non-discrimination and equity in healthcare, should be a key requirement for the widespread implementation of AI algorithms. However, it is not yet specifically mandated by European legislation (Regulation 2017/745) and this not a prerequisite for marketing an AI algorithm^28^. For this reason, different groups of experts around the world have developed guidelines to stipulate the essential requirements for using AI algorithms as a complementary diagnostic tool. Yet, while expert groups emphasise the necessity of external validation to confirm its practical potential in clinical settings, this requirement is not yet mandated in the Regulation^26,29,30^.

The implementation of AI in healthcare appears to be an imminent reality that can offer significant benefits to both professionals and the general population. However, it is essential to implement safe and validated tools in real clinical settings to maintain fairness. In this sense, this study aims to externally validate an AI algorithm's diagnoses in real primary care settings, comparing them to a radiologist’s diagnoses. The aim is also to identify diagnoses the algorithm may not have been trained for.

## Materials and methods

The study protocol has been previously described and published^32^. Nevertheless, the most relevant points of the present study are described below.

### The ChestEye AI algorithm

Oxipit^33^, one of the leading companies in AI medical image reading, has developed a fully automatic computer-aided diagnosis (CAD) AI algorithm for reading chest X-rays trained with more than 300,000 images, available through a web platform called ChestEye. The ChestEye imaging service has been certified as a Class II medical device on the Australian Register of Therapeutic Goods and has also been CE marked^33^. The web platform reads the inserted chest X-ray and returns the automatic report with the ability to detect 75 conditions, which cover 90% of diagnoses, as well as a heat map to show the locations of the findings. Thus, ChestEye allows radiologists to analyse only the most relevant X-rays^33,34^.

### Study design

A prospective observational study for the external validation of the AI algorithm in a region of Catalonia of users who were scheduled for chest radiography at the Osona Primary Care Center. For each user, the report of the reference radiologist (considered the gold standard) was obtained. Subsequently, the research team input the image into the AI algorithm to obtaine the diagnosis. This allowed for the comparison of the AI's performance with the reference standard in terms of accuracy, sensitivity, specificity, positive predictive value and negative predictive value c.

### Description of the study population, time frame, and data collection

The study was carried out at the Catalan Institute of Health’s Primary Care Centre Vic Nord (Osona, Catalonia, Spain), a reference centre where all chest X-rays in the region are performed (with a coverage of 125,000 users). At this same centre, convenience recruitment was carried out from 7 February 2022 to 31 May 2022. The study was explained, and the information sheet and informed consent were given to all patients who came for a chest X-ray and met the inclusion criteria^32^.

The reference population of the study was the entire population of the Osona region who underwent chest X-rays at the study centre and agreed to participate in the study. The study included only anteroposterior chest X-rays on those over 18 years of age and excluded pregnant women and poor-quality chest X-rays (poor exposure, non-centred or rotated images).

### Sample size

Due to a problem with the image collection centre, the sample size calculated in the protocol^32^ could not be reached. For this reason, the sample was recalculated by increasing the precision by one percentage point. Thus, in order to validate the algorithm, a sample of 450 images was needed to estimate an overall accuracy expected to be around 80%, with a confidence interval of 95%, a precision level of 5% and a percentage of replacements needed of 15%.

### Procedure

Once recruitment was completed, the Technical Service of the Catalan Institute of Health of Central Catalonia extracted the patients’ anonymous and automated images and their corresponding non-anonymised reports. The images and reports were then coded together so that they could be related.

The research team then entered all the images into the AI model to extract their interpretation (the diagnosis or the possibility of no abnormalities). At the same time, three general practitioners interpreted the reference radiologists’ reports, without seeing the images in order to avoid assumptions, with the aim of extracting the diagnoses described.

Finally, the group of general practitioners grouped all the conditions detectable by the AI model into 9 categories according to the anatomy of the thorax in order to build an individual and grouped study. The categories were: external implants, mediastinal findings or conditions, cardiac and valvular conditions, vessel conditions, bone conditions, pleural or pleural space conditions, upper abdominal findings or conditions, pulmonary parenchymal findings or conditions, and others.

### Statistical analysis

To validate the algorithm, the AI algorithm’s diagnoses were compared with those of the gold standard. The accuracy of the algorithm and the confusion matrix were obtained from the images correctly classified positive (PT), correctly classified negative (TN), false positive (FP) and false negative (FN). Sensitivity and specificity were also calculated. These measurements were obtained for the total sample, for each condition and for each of the categories according to physiology. Analyses were performed with R software version 4.2.1 and all confidence intervals were 95%.

### Ethics committee

The University Institute for Research in Primary Health Care Jordi Gol i Gurina (Barcelona, Spain) ethics committee approved the trial study protocol (approval code: 21/288). Written informed consent was requested from all patients participating in the study.

### Ethical considerations

Radiologists’ assessment and decisions were not influenced by this study, as the normal radiology referral workflow was not affected. This project was approved by the Research Ethics Committee (REC) from the Foundation University Institute for Primary Health Care Research Jordi Gol i Gurina (IDIAPJGol) (P21/288-P). The study was performed in accordance with relevant guidelines/regulations, and informed consent was obtained from all participants. All research was performed in accordance with the Declaration of Helsinki.

## Results

**Figure 1:**
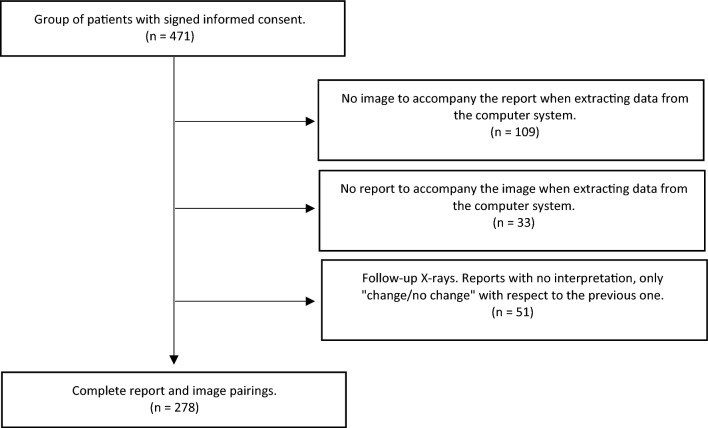
Flow chart of the final sample of study images.

Of the 471 patients who agreed to participate in the study and provide the images and reports, the final sample for external validation of the model was 278, mainly due to computer-related issues when extracting the data. In some cases, when automatically extracting images and reports, both were not obtained, i.e., either the image was missing, or the report was missing. In addition, some reports did not include the interpretation of the image, as it was a follow-up X-ray. In these cases, the report only indicated whether or not there were changes with respect to the previous report and, therefore, they had to be discarded from the analysis (Fig. 1). Of the final sample, 144 (51.8%) obtained images without radiological abnormalities according to the radiologist's report.

Although the final sample consisted of 278 images, it is possible that an image may be suggestive of one or more conditions or anatomical abnormalities. In this sense, the sum of the unaltered images plus the images with one or more abnormalities does not correspond to the total sample (n = 278). Of the conditions or anatomical abnormalities for which the algorithm was trained, the AI model identified 33 in the sample, and the most prevalent were: nodule (n = 37, 13.3%), consolidation (n = 28, 10.1%), abnormal rib (n = 17, 6.1%), enlarged heart (n = 15, 5.4%) and aortic sclerosis (n = 8, 2.9%). On the other hand, the reference radiologist identified 35 in the sample, and the most prevalent were: consolidation (n = 30, 10.8%), pulmonary emphysema (n = 20, 7.2%), enlarged aorta (n = 14, 5.0%), enlarged heart (n = 12, 4.3%), hilar prominence (n = 12, 4.3%) and linear atelectasis (n = 11, 3.9%) (Table 1).

**Table 1:** Description of the conditions or anatomical abnormalities of the 278 images and their respective diagnoses according to the radiologist and the AI algorithm.

Condition/finding	Radiologist N (%)	AI algorithm N (%)
Pleural adhesion	–	5 (1.8)
Enlarged aorta	14 (5.04)	1 (0.36)
Linear atelectasis	11 (3.96)	4 (1.44)
Nuss bar or Pectus excavatum	1 (0.36)	–
Sternal wires	3 (1.08)	3 (1.08)
Lymph node calcification	–	2 (0.72)
Spinal degenerative changes	6 (2.16)	–
Enlarged heart	12 (4.32)	15 (5.4)
Kyphosis	3 (1.08)	–
Catheter placement	1 (0.36)	–
Congestion	–	3 (1.08)
Consolidation	30 (10.79)	28 (10.07)
Abnormal rib	3 (1.08)	17 (6.12)
Mediastinal shift	–	1 (0.36)
Hilar prominence	12 (4.32)	–
Elevated diaphragm	4 (1.44)	7 (2.52)
Pulmonary emphysema	20 (7.19)	3 (1.08)
Bullous emphysema	2 (0.72)	–
Pleural thickening	4 (1.44)	–
Fissural thickening	1 (0.36)	2 (0.72)
Spinal enthesopathy	1 (0.36)	–
Aortic sclerosis	2 (0.72)	8 (2.88)
Scoliosis	3 (1.08)	3 (1.08)
Pulmonary fibrosis	1 (0.36)	–
Spinal fracture	6 (2.16)	–
Gastric bubble	–	3 (1.08)
Granuloma	2 (0.72)	4 (1.44)
Hiatal hernia	5 (1.8)	6 (2.16)
Pulmonary hypertension	3 (1.08)	–
Hypoventilation	–	1 (0.36)
Spinal implant	1 (0.36)	1 (0.36)
Lymphadenopathy	2 (0.72)	5 (1.8)
Pacemaker	2 (0.72)	2 (0.72)
Interstitial markings	4 (1.44)	8 (2.88)
Mass	1 (0.36)	–
Widened mediastinum	1 (0.36)	1 (0.36)
Pneumoperitoneum	–	1 (0.36)
Nodule	3 (1.08)	37 (13.31)
Pneumomediastinum	–	3 (1.08)
Pneumothorax	–	1 (0.36)
Sarcoidosis	–	4 (1.44)
Tuberculosis	1 (0.36)	6 (2.16)
Artificial heart valve	1 (0.36)	1 (0.36)
Pleural effusion	10 (3.6)	6 (2.16)
No abnormalities	144 (51.8)	203 (73.02)
Others	63 (22.67)	–

Analysing the validity of the AI algorithm, the average accuracy was 0.95 (95% CI 0.92; 0.98), the average sensitivity was 0.48 (95% CI 0.30; 0.66) and the average specificity was 0.98 (95% CI 0.97; 0.99). The accuracy, sensitivity and specificity values for each condition can be seen in Table 2. The values for true positives, true negatives, false positives, and false negatives for each condition are presented in Table 1 of the supplementary information.

**Table 2:** Accuracy, sensitivity, specificity, positive and negative predictive values with a 95% confidence interval for each condition.

Condition/finding	Accuracy	95% CI	Sensitivity	95% CI	Specificity	95% CI	PPV	95% CI	NPV	95% CI
Average	0.95	(0.92; 0.98)	0.48	(0.30; 0.66)	0.98	(0.97; 0.99)				
Pleural adhesion	–	–	–	–	–	–	–	–	–	–
Enlarged aorta	0.95	(0.92; 0.97)	0.07	(0.00; 0.34)	1.00	(0.99; 1.00)	1.00	(0.02; 1.00)	0.95	(0.92; 0.97)
Linear atelectasis	0.70	(0.94; 0.98)	0.27	(0.06; 0.61)	0.99	(0.98; 1.00)	0.75	(0.19; 0.99)	0.97	(0.94; 0.99)
Nuss bar or Pectus excavatum	–	–	–	–	–	–	–	–	–	–
Sternal wires	1.00	(0.98; 1.00)	1.00	(0.29; 1.00)	1.00	(0.99; 1.00)	1.00	(0.29; 1.00)	1.00	(0.99; 1.00)
Lymph node calcification	–	–	–	–	–	–	–	–	–	–
Spinal degenerative changes	–	–	–	–	–	–	–	–	–	–
Enlarged heart	0.96	(0.93; 0.98)	0.67	(0.35; 0.90)	0.97	(0.95; 0.99)	0.53	(0.27; 0.79)	0.98	(0.96; 1.00)
Kyphosis	–	–	–	–	–	–	–	–	–	–
Catheter placement	–	–	–	–	–	–	–	–	–	–
Congestion	–	–	–	–	–	–	–	–	–	–
Consolidation	0.89	(0.86; 0.93)	0.50	(0.31; 0.69)	0.95	(0.91; 0.97)	0.54	(0.34; 0.72)	0.94	(0.90; 0.97)
Abnormal rib	0.94	(0.91; 0.97)	0.67	(0.09; 0.99)	0.95	(0.91; 0.97)	0.12	(0.01; 0.36)	0.99	(0.98; 1.00)
Mediastinal shift	–	–	–	–	–	–	–	–	–	–
Hilar prominence	–	–	–	–	–	–	–	–	–	–
Elevated diaphragm	0.97	(0.94; 0.98)	0.25	(0.01; 0.81)	0.98	(0.95; 0.99)	0.14	(0.00; 0.58)	0.99	(0.97; 1.00)
Pulmonary emphysema	0.93	(0.89; 0.96)	0.10	(0.01; 0.32)	0.99	(0.98; 1.00)	0.67	(0.09; 0.99)	0.93	(0.90; 0.96)
Bullous emphysema	–	–	–	–	–	–	–	–	–	–
Pleural thickening	–	–	–	–	–	–	–	–	–	–
Fissural thickening	0.99	(0.98; 0.99)	1.00	(0.03; 1.00)	0.99	(0.98; 1.00)	0.50	(0.01; 0.99)	1.00	(0.99; 1.00)
Spinal enthesopathy	–	–	–	–	–	–	–	–	–	–
Aortic sclerosis	0.96	(0.93; 0.98)	0.00	(0.00; 0.37)	0.97	(0.94; 0.99)	0.00	(0.00; 0.37)	0.99	(0.97; 1.00)
Scoliosis	0.98	(0.96; 0.99)	0.33	(0.01; 0.91)	0.99	(0.97; 1.00)	0.33	(0.01; 0.91)	0.99	(0.97; 1.00)
Pulmonary fibrosis	–	–	–	–	–	–	–	–	–	–
Spinal fracture	–	–	–	–	–	–	–	–	–	–
Gastric bubble	–	–	–	–	–	–	–	–	–	–
Granuloma	0.98	(0.95; 0.99)	0.00	(0.00; 0.84)	0.98	(0.96; 1.00)	0.00	(0.00; 0.60)	0.99	(0.97; 1.00)
Hiatal hernia	0.99	(0.98; 0.99)	1.00	(0.48; 1.00)	0.99	(0.98; 1.00)	0.83	(0.36; 1.00)	1.00	(0.99; 1.00)
Pulmonary hypertension	–	–	–	–	–	–	–	–	–	–
Hypoventilation	–	–	–	–	–	–	–	–	–	–
Spinal implant	1.00	(0.98; 1.00)	1.00	(0.03; 1.00)	1.00	(0.99; 1.00)	1.00	(0.03; 1.00)	1.00	(0.99; 1.00)
Lymphadenopathy	0.97	(0.95; 0.99)	0.50	(0.00; 0.97)	0.98	(0.96; 0.99)	0.20	(0.00; 0.52)	0.99	(0.98; 1.00)
Pacemaker	0.99	(0.97; 0.99)	0.50	(0.01; 0.99)	0.99	(0.98; 1.00)	0.50	(0.01; 0.99)	0.99	(0.98; 1.00)
Interstitial markings	0.98	(0.95; 0.99)	0.75	(0.19; 0.99)	0.98	(0.96; 0.99)	0.38	(0.09; 0.76)	0.99	(0.98; 1.00)
Mass	–	–	–	–	–	–	–	–	–	–
Widened mediastinum	0.99	(0.97; 0.99)	0.00	(0.00; 0.97)	0.99	(0.98; 1.00)	0.00	(0.00; 0.97)	0.99	(0.98; 1.00)
Pneumoperitoneum	–	–	–	–	–	–	–	–	–	–
Nodule	0.86	(0.82; 0.91)	0.00	(0.00; 0.71)	0.88	(0.84; 0.92)	0.00	(0.00; 0.11)	0.99	(0.96; 1.00)
Pneumomediastinum	–	–	–	–	–	–	–	–	–	–
Pneumothorax	–	–	–	–	–	–	–	–	–	–
Sarcoidosis	–	–	–	–	–	–	–	–	–	–
Tuberculosis	0.98	(0.96; 0.99)	1.00	(0.03; 1.00)	0.98	(0.96; 0.99)	0.17	(0.00; 0.64)	1.00	(0.99; 1.00)
Artificial heart valve	1.00	(0.98; 1.00)	1.00	(0.03; 1.00)	1.00	(0.99; 1.00)	1.00	(0.02; 1.00)	1.00	(0.99; 1.00)
Pleural effusion	0.97	(0.94; 0.98)	0.40	(0.12; 0.74)	0.99	(0.97; 1.00)	0.67	(0.22; 0.96)	0.98	(0.95; 0.99)
Others	–	–	–	–	–	–	–	–	–	–

*PPV* positive predictive value; *NVP* negative predictive value.

The reference radiologist identified a list of conditions for which the algorithm was not trained, and which were therefore classified as “Other”. The most prevalent were bronchial wall thickening (n = 13, 4.68%), fibroscarring lesions or abnormalities (n = 11, 3.96%) and chronic pulmonary abnormalities (n = 11, 3.96%) (Table 3).

**Table 3:** Description of conditions not contemplated by the AI model.

Condition/finding	N (%)
Air bronchogram	1 (0.36)
Chronic bronchopathy/COPD	4 (1.44)
Bronchiectasis	6 (2.16)
Aortic calcification	1 (0.36)
Post-surgical abnormalities	4 (1.44)
Chronic pulmonary abnormalities	11 (3.96)
Chondrocalcinosis	1 (0.36)
Bronchial wall thickening	13 (4.68)
Ankylosing spondylitis	1 (0.36)
Nonspecific	2 (0.72)
Fibrocystic lesions or fibrocystic abnormalities	11 (3.96)
Diaphragmatic eventration	6 (2.16)
Breast prostheses	1 (0.36)
Linear opacities	1 (0.36)

**Figure 2:**
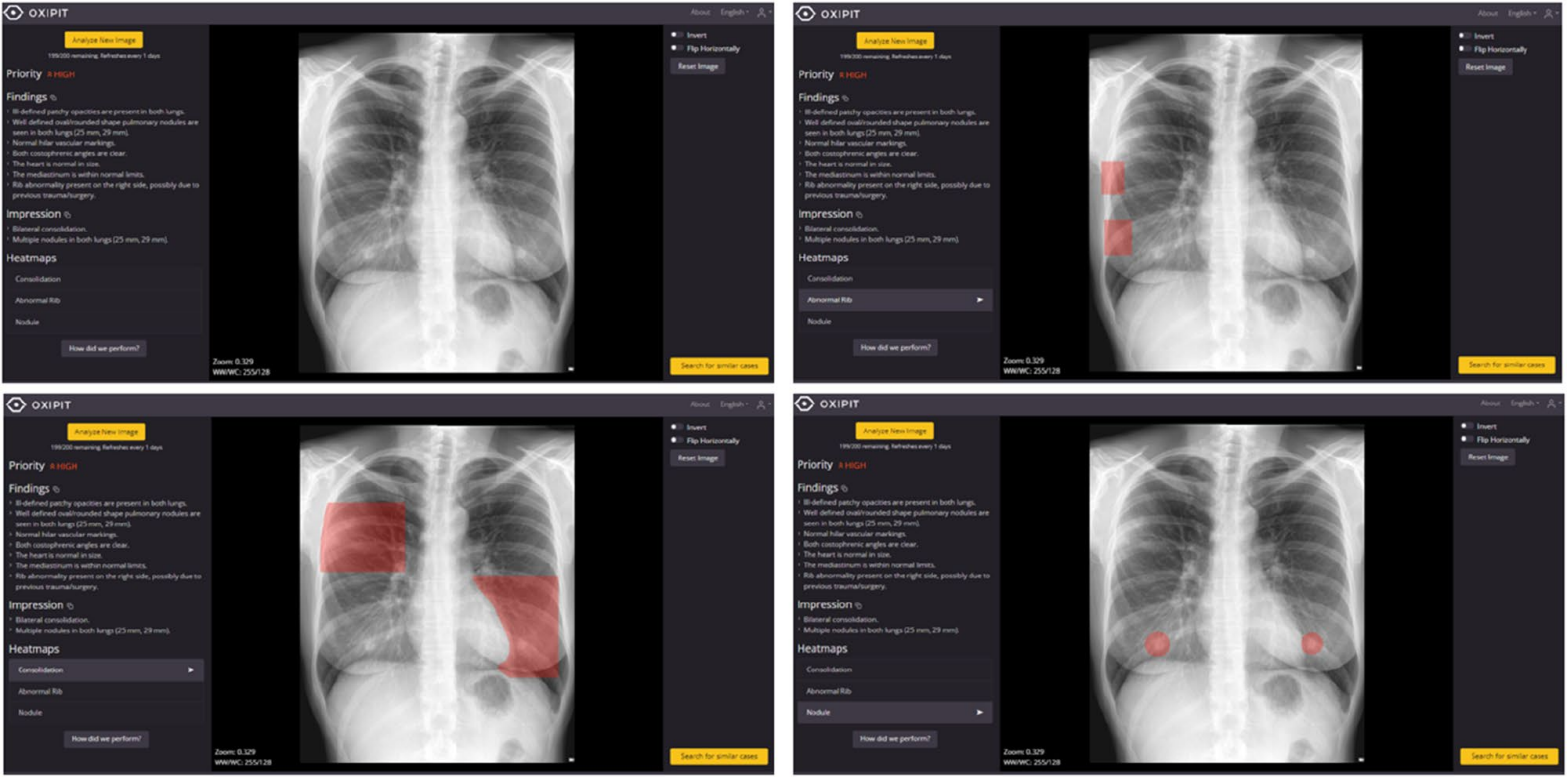
Image of patient (upper-left) where according to the radiologist's report there is only consolidation, but the algorithm detects an abnormal rib (upper-right), consolidation (lower-left) and two nodules (lower-right). It is worth noting the confusion of a consolidation with mammary tissue and of two nodules with the two mammary areolae.

**Figure 3:**
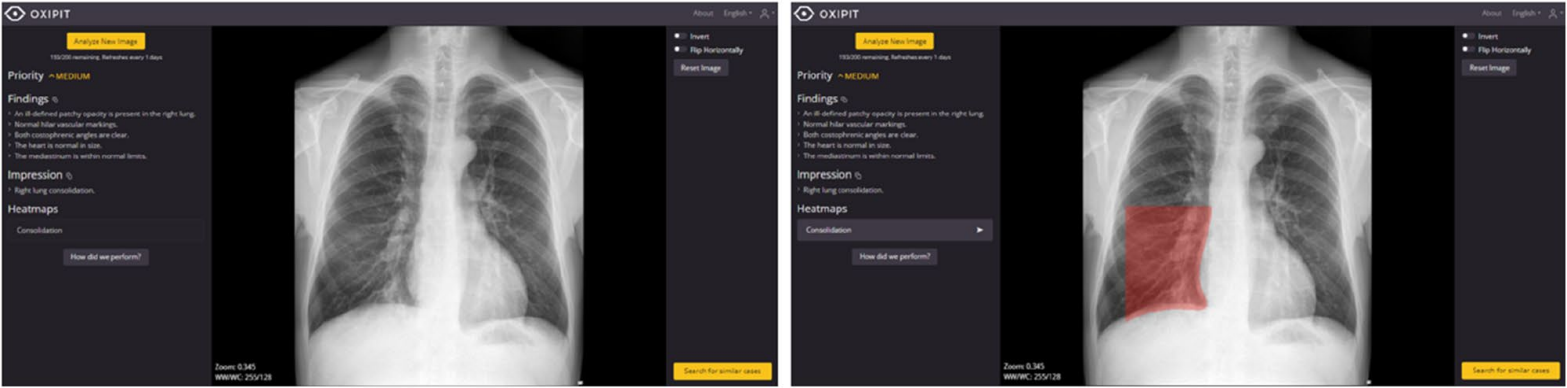
Image of patient (left) where the AI algorithm and the radiologist detected the same condition: consolidation (right).

Figures 2 and 3 show some examples of the AI algorithm’s performance in cases where the algorithm’s diagnosis was successful and in cases where errors occurred.

In order to perform a more general analysis, the conditions were grouped into 10 groups according to chest anatomy, considering only the diagnoses for which the AI model was trained. According to the radiologist, the most prevalent groupings found were lung parenchymal conditions (n = 71, 25.5%), bone conditions (n = 21, 7.5%) and vessel conditions (n = 18, 6.47%). According to the AI model, the most prevalent conditions were lung parenchymal conditions (n = 57, 20.5%), bone conditions (n = 21, 7.5%) and cardiac and/or valvular conditions (n = 15, 5.4%) (Table 4).

**Table 4:** Description of the conditions of the 278 images according to the radiologist and AI model, grouped according to chest anatomy.

Grouping	Radiologist N (%)	AI algorithm N (%)
Others	3 (1.08)	9 (3.24)
External implants	6 (2.16)	5 (1.80)
Mediastinum	1 (0.36)	4 (1.44)
Upper abdomen conditions	9 (3.24)	13 (4.68)
Cardiac and/or valvular conditions	12 (4.32)	15 (5.40)
Vessel conditions	18 (6.47)	9 (3.24)
Bone conditions	21 (7.55)	21 (7.55)
Pulmonary parenchymal conditions	71 (25.50)	57 (20.5)
Pleural conditions	13 (4.68)	12 (4.32)
No abnormalities	144 (51.8)	203 (73.0)

Finally, Table 5 shows the accuracy, sensitivity and specificity values for each group. Of these, it is worth mentioning the low sensitivity values for mediastinal conditions (0.0, 95% CI 0.0; 0.96), vessel conditions (0.29, 95% CI 0.11; 0.52) and bone conditions (0.24, 95% CI 0.08; 0.47). On the other hand, high sensitivity values were recorded for external implants (0.67, 95% CI 0.22; 0.96), upper abdominal conditions (0.67, 95% CI 0.30; 0.93) and cardiac and/or valvular conditions (0.67, 95% CI 0.35; 0.90). It is also worth mentioning the model’s strong ability to detect images that do not have radiological abnormalities. The values for true positives, true negatives, false positives, and false negatives for each grouping are presented in Table 1 of the supplementary information.

**Table 5:** Accuracy, sensitivity and specificity values for each grouping.

Condition/finding	Accuracy	95% CI	Sensitivity	95% CI	Specificity	95% CI
Others	0.96	(0.93; 0.98)	0.33	(0.01; 0.91)	0.97	(0.94; 0.99)
External implants	0.99	(0.97; 0.99)	0.67	(0.22; 0.96)	0.99	(0.98; 1.00)
Mediastinum	0.98	(0.96; 0.99)	0.00	(0.00; 0.97)	0.99	(0.96; 1.00)
Upper abdomen conditions	0.96	(0.93; 0.98)	0.67	(0.30; 0.93)	0.97	(0.95; 0.99)
Cardiac and/or valvular conditions	0.96	(0.93; 0.98)	0.67	(0.35; 0.90)	0.97	(0.95; 0.99)
Vessel conditions	0.92	(0.89; 0.95)	0.29	(0.11; 0.52)	0.99	(0.97; 1.00)
Bone conditions	0.89	(0.84; 0.92)	0.24	(0.08; 0.47)	0.94	(0.90; 0.96)
Pulmonary parenchymal conditions	0.78	(0.72; 0.82)	0.46	(0.35; 0.59)	0.88	(0.83; 0.92)
Pleural conditions	0.95	(0.92; 0.97)	0.46	(0.19; 0.75)	0.98	(0.95; 0.99)
With abnormalities VS without	0.70	(0.64; 0.75)	0.47	(0.38; 0.56)	0.92	(0.86; 0.96)

## Discussion

The aim of this study was to perform an external validation, in real clinical practice, of the diagnostic capability of an AI algorithm with respect to the reference radiologist for chest X-rays, as well as to detect possible diagnoses for which the algorithm had not been trained. Thus, the overall accuracy of the algorithm was 0.95 (95% CI 0.92–0.98), the sensitivity was 0.48 (95% CI 0.30–0.66) and the specificity was 0.98 (95% CI 0.97–0.99). The results obtained have further highlighted, as indicated by different expert groups^26,28,29^, the need for external validations of AI algorithms in a real clinical context in order to establish the necessary measures and adaptations to ensure safety and effectiveness in any environment. Therefore, in the context of the model developed, it is important to understand and interpret what each of the results obtained indicate.

High accuracy values were observed in most cases (ranging between 0.7–1). The accuracy is represented by the proportion of correctly classified results among the total number of cases examined. This value was high since, both for each condition and for the groups of conditions, the capacity to detect true negatives was good, taking into account that most of the images analysed were found to have no abnormalities (51.8%). Working with an AI algorithm that quickly determines that there is no abnormality can function as a triage tool, streamlining the diagnostic process, allowing the professional to focus on other tests, reduce waiting lists, reduce waiting times for diagnoses and even reduce expenses in secondary tests.

With sensitivity referring to the ability to detect an abnormality when there really is one, high sensitivity values were shown when detecting anatomical findings or abnormalities such as sternal cables, enlarged heart, abnormal ribs, spinal implants, cardiac valve, or interstitial markings. On the other hand, low sensitivity values were observed for most conditions, indicating that the algorithm had limited ability to detect certain conditions like those in the mediastinum, vessels, or bones. These findings align with the results of a study that performed an external validation of a similar algorithm in an emergency department^35^. Additionally, the algorithm exhibited low sensitivity in detecting pulmonary emphysema, linear atelectasis, and hilar prominence, which are prevalent conditions in the primary care setting^31^.

Low sensitivity was also observed when detecting nodules, with the algorithm finding more nodules than the reference radiologist, in most cases confusing them with areolae in the breast tissue. Although it is important to be able to detect any warning signs and that the professional is in charge of making the clinical judgement and determining the need for complementary tests, it is possible that this external validation has detected a possible gender bias in the training of the algorithm. When it comes to chest imaging, it's important to distinguish between the physiological aspects of breast tissue and any potential changes it may undergo during various life stages, as opposed to signs of conditions or abnormalities^36^. Other studies have also detected a high false positive value in the detection of nodules due to other causes such as fat, pleura or interstitial lung disease^37^.

Finally, specificity being the ability to correctly identify images in which there are no radiological abnormalities, the results showed high values for all condition groupings, since the algorithm was able to detect images with no abnormalities.

Following the authors' desire to contribute to the improvement of the AI model, some radiologists’ findings were identified that were overlooked during the algorithm's training, especially related to bronchial conditions, including chronic bronchopathy, bronchiectasis, and bronchial wall thickening. Additionally, the algorithm missed common chronic conditions often seen in primary care, including chronic pulmonary abnormalities, COPD, and fibrocystic abnormalities. Furthermore, it was noted that certain condition names within the AI algorithm should be adjusted to align with names used in the radiology field. Interstitial markings could be changed to interstitial abnormality, consolidation to condensation, aortic sclerosis to valvular sclerosis, and abnormal rib to rib fracture.

Once the main variables that characterise the algorithm's capacity were discussed, the results obtained differ from the majority of published studies, since most of them obtained a higher algorithm capacity. However, it should be noted that most of these are internal validations and not tested in real clinical practice settings^38–40^.

A study in Korea performed an internal and external validation of an AI algorithm capable of detecting the 10 most prevalent chest X-ray abnormalities and was able to demonstrate the difference in sensitivity and specificity values. The internal validation obtained sensitivity and specificity values between 0.87–0.94 and 0.81–0.98, respectively. On the other hand, the external validation obtained sensitivity and specificity values between 0.61–1.00 and 0.71–0.98, respectively^41^. This difference can also be seen in a study in Michigan, where internal and external validation of an AI algorithm capable of detecting the most common chest X-ray abnormalities was performed^42^, and in a study at the Seoul University School of Medicine, where an algorithm for lung cancer detection in population screening was validated^43^.

Therefore, the results obtained from the external validation show the need to increase the sensitivity of the algorithm for most conditions. Considering that AI should serve as a diagnostic support tool and the ultimate responsibility for medical decisions rests with the practitioner, it is ideal for the algorithm to flag potential abnormalities for the practitioner to review and confirm. This ensures the highest diagnostic effectiveness. Recent studies have shown that the use of an AI algorithm to support the practitioner significantly improves diagnostic sensitivity and specificity and reduces image reading time^20,44^.

Enhanced sensitivity could help address the shortage of specialised radiologists globally, especially in Central Catalonia's primary care setting, where this validation was conducted^45,46^. More and more, general practitioners are tasked with interpreting X-rays. In this context, the advancement of these tools can be a valuable asset in the diagnostic process.

## Limitations and strengths

One significant limitation of the study was the small sample size for certain specific conditions. This was due to difficulties in obtaining the required number of cases, as these conditions are not very common in real clinical practice. Consequently, the external validation for these conditions yielded less reliable estimates. However, by representing reality, a large volume of images without radiological abnormalities was obtained and this allowed for a good external validation of the model's ability to detect images without abnormalities.

In addition, the radiologist's reference diagnosis was not always the practitioner’s own, but that of a group of radiologists. This could represent a limitation, since there was no consensus among them, but there was no desire to alter actual clinical practice. In addition, the study aimed to test the algorithm in primary care settings. For this reason, a double interpretation of the images was performed: initially by the radiologist and subsequently, the radiologist's report was interpreted by the family and community physician.Finally, another limitation was the lack of information on the sex of the users analysed. Through the results obtained, we found it very relevant to do another study but separating the capabilities of the algorithm according to gender, since it seems that they might not be the same. In addition, since we have a small sample for most of the conditions, separating the analyses according to sex in the present study would be unreliable and not comparable.

On the other hand, the greatest strength of the study is that it presents an external validation in real clinical practice in primary care and there are currently few studies that have done so. Most studies present an internal validation, but it is very important to perform an external validation in order to estimate the accuracy of the model in a population other than the training population, thus allowing the results to be generalised.

## Conclusion

The findings of this study demonstrate the validation of an AI algorithm for reading chest X-rays in the primary care setting, achieved by comparing its diagnoses with those made by a radiologist. The algorithm has been validated in the primary care setting using values such as the accuracy, sensitivity and specificity of the algorithm and has proven to be useful by being able to identify images with or without abnormalities. However, further training is needed to increase the diagnostic capability of some of the conditions analysed. It is important that training is done in a real environment, with real images, in order to perform robust external validations. Our analysis highlights the need for continuous improvement to ensure that the algorithm is a reliable and effective tool in the primary care environment.

The role of AI in healthcare should be to assist and support the practitioners. Being able to reliably detect images without abnormalities can have a very positive impact, reducing waiting times for diagnoses, secondary tests to rule out conditions, streamlining practitioners work and, among others, ultimately favouring patient care and, indirectly, their health.

### Supplementary Information

Supplementary Table 1.

## Data Availability

The datasets generated and/or analysed during the current study are not publicly available because our manuscript was based on confidential and sensitive health data. However, they are available from the corresponding author upon reasonable request.
