# Supplementary material for: Real-world testing of an artificial intelligence algorithm for the analysis of chest X-rays in primary care settings
Source: Sci Rep. 2024 Mar 3;14:5199. doi: 10.1038/s41598-024-55792-1 (PMC10908781; doi:10.1038/s41598-024-55792-1)
Supplement: Supplementary file 1 — Supplementary Table 1. [file 41598_2024_55792_MOESM1_ESM.docx]

Table 1. Confusion matrix values for each condition and for groupings.

| **Condition/finding** | **TP** | **TN** | **FP** | **FN** |
| --- | --- | --- | --- | --- |
| Pleural adhesion | - | - | - | - |
| Enlarged aorta | 1 | 264 | 0 | 13 |
| Linear atelectasis | 3 | 266 | 1 | 8 |
| Nuss bar or Pectus excavatum | - | - | - | - |
| Sternal wires | 3 | 275 | 0 | 0 |
| Lymph node calcification | - | - | - | - |
| Spinal degenerative changes | - | - | - | - |
| Enlarged heart | 8 | 259 | 7 | 4 |
| Kyphosis | - | - | - | - |
| Catheter placement | - | - | - | - |
| Congestion | - | - | - | - |
| Consolidation | 15 | 235 | 13 | 15 |
| Abnormal rib | 2 | 260 | 15 | 1 |
| Mediastinal shift | - | - | - | - |
| Hilar prominence | - | - | - | - |
| Elevated diaphragm | 1 | 268 | 6 | 3 |
| Pulmonary emphysema | 2 | 257 | 1 | 18 |
| Bullous emphysema | - | - | - | - |
| Pleural thickening | - | - | - | - |
| Fissural thickening | 1 | 276 | 1 | 0 |
| Spinal enthesopathy | - | - | - | - |
| Aortic sclerosis | 0 | 268 | 8 | 2 |
| Scoliosis | 1 | 273 | 2 | 2 |
| Pulmonary fibrosis | - | - | - | - |
| Spinal fracture | - | - | - | - |
| Gastric bubble | - | - | - | - |
| Granuloma | 0 | 272 | 4 | 2 |
| Hiatal hernia | 5 | 272 | 1 | 0 |
| Pulmonary hypertension | - | - | - | - |
| Hypoventilation | - | - | - | - |
| Spinal implant | 1 | 277 | 0 | 0 |
| Lymphadenopathy | 1 | 272 | 4 | 1 |
| Pacemaker | 1 | 275 | 1 | 1 |
| Interstitial markings | 3 | 269 | 5 | 1 |
| Mass | - | - | - | - |
| Widened mediastinum | 0 | 276 | 1 | 1 |
| Pneumoperitoneum | - | - | - | - |
| Nodule | 0 | 242 | 33 | 3 |
| Pneumomediastinum | - | - | - | - |
| Pneumothorax | - | - | - | - |
| Sarcoidosis | - | - | - | - |
| Tuberculosis | 1 | 272 | 5 | 0 |
| Artificial heart valve | 1 | 277 | 0 | 0 |
| Pleural effusion | 4 | 266 | 2 | 6 |
|  |  |  |  |  |
| Others | 2 | 267 | 7 | 2 |
| External implants | 4 | 271 | 1 | 2 |
| Mediastinum | 0 | 273 | 4 | 1 |
| Upper abdomen conditions | 6 | 262 | 7 | 3 |
| Cardiac and/or valvular conditions | 8 | 259 | 7 | 4 |
| Vessel conditions | 3 | 254 | 6 | 15 |
| Bone conditions | 5 | 241 | 16 | 16 |
| Pulmonary parenchymal conditions | 33 | 183 | 24 | 38 |
| Pleural conditions | 6 | 259 | 6 | 7 |
| No abnormalities | 132 | 63 | 71 | 12 |

*TP: True positive, TN: True negative, FP: False positive and FN: False negative.*
